# The Probiotic Mixture VSL#3 Dampens LPS-Induced Chemokine Expression in Human Dendritic Cells by Inhibition of STAT-1 Phosphorylation

**DOI:** 10.1371/journal.pone.0115676

**Published:** 2014-12-29

**Authors:** Rob Mariman, Frans Tielen, Frits Koning, Lex Nagelkerken

**Affiliations:** 1 Department of Metabolic Health Research, TNO, Leiden, The Netherlands; 2 Department of Immunohematology and Bloodtransfusion, Leiden University Medical Centrum, Leiden, The Netherlands; University of Leuven, Rega Institute, Belgium

## Abstract

VSL#3, a mixture of 8 different probiotic bacteria, has successfully been used in the clinic to treat Ulcerative Colitis. We previously identified the modulation of chemokines as a major mechanism in the protective effect of the VSL#3 in a mouse model of colitis. This was supported by *in* *vitro* studies that implicated a role for VSL#3 in the suppression of LPS-induced chemokine production by mouse bone marrow-derived dendritic cells (DC). Herein, we validated these findings employing human monocyte-derived DC. Stimulation of human DC with LPS, VSL#3, or a combination of both resulted in their maturation, evident from enhanced expression of activation markers on the cell-surface, as well as the induction of various chemokines and cytokines. Interestingly, a set of LPS-induced chemokines was identified that were suppressed by VSL#3. These included CXCL9, CXCL10, CCL2, CCL7, and CCL8. *In silico* approaches identified STAT-1 as a dominant regulator of these chemokines, and this was confirmed by demonstrating that LPS-induced phosphorylation of this transcription factor was inhibited by VSL#3. This indicates that VSL#3 may contribute to the control of inflammation by selective suppression of STAT-1 induced chemokines.

## Introduction

The gastrointestinal tract is continuously exposed to foreign antigens – mainly derived from the commensal microflora and/or food antigens [1]. Immune responses in the intestinal mucosa need to be tightly regulated by activating pro-inflammatory pathways for appropriate host defense against pathogenic microorganisms while remaining unresponsive to symbiotic bacteria [2], [3]. Under homeostasis conditions little or no inflammation occurs in the gut associated lymphoid tissue (GALT). However, genetic defects and impairment of barrier integrity may cause exaggerated immune responses driven by the microflora resulting in the development of intestinal inflammation [4]. Specific lactobacilli and bifidobacteria have been shown efficient in modulating intestinal immunity in homeostasis [5] and conditions of chronic intestinal inflammation like inflammatory bowel disease [6]. Several modes of action of probiotic bacteria have been identified, including restoration of microbial homeostasis through microbe-microbe interactions, pathogen inhibition, enhancement of barrier integrity, or via direct modulation of immune responses [7].

In the gastro-intestinal tract, probiotic bacteria may be recognized by a plethora of pattern recognition receptors, including toll-like receptors, C-type lectin receptors and NOD-like receptors on epithelial cells and innate immune cells, such as dendritic cells (DC) [8], [9]. Maturation of DC after contact with antigen or inflammatory stimuli is accompanied by functional and phenotypic changes. Dependent on the stimulus, DC can secrete chemokines and cytokines, thereby inducing naïve T cell proliferation and polarization towards Th1, Th2, Th17 effector cells or regulatory T cells [10]. Accordingly, much attention has focused on the impact of DC priming by probiotic bacteria to modulate adaptive immune responses. We previously identified the modulation of chemokines derived from innate immune cells, like DC, as a major mechanism in the protective effect of the VSL#3 in a mouse model of colitis. This was supported by *in* *vitro* studies that implicated a role for VSL#3 in the suppression of LPS-induced chemokine production by mouse bone marrow derived dendritic cells (26). We aimed to translate these finding to the human situation, and therefore studied modulation of human monocyte-derived DC by a mixture of VSL#3 in response to the TLR-4 agonist LPS and evaluated chemokine mRNA expression and protein secretion. By this approach we identified mechanisms by which VSL#3 interferes with the secretion of pro-inflammatory chemokines.

## Materials and Methods

### DC cultures

Human peripheral blood mononuclear cells (PBMC) were isolated from buffy coats of healthy donors (Sanquin, Leiden, the Netherlands) by Histopaque (Sigma Diagnostics St.Louis, MO, USA) density gradient centrifugation. Human monocytes were purified from PBMC by positive selection using anti-CD14-conjugated magnetic microbeads (StemCell Technologies, Grenoble, France). The recovered cells were 95% to 99% CD14^+^ as determined by flow cytometry. Monocytes were cultured at 2×10^6^/ml in RPMI1640 supplemented with 100 U/ml penicillin, 100 µg/ml streptomycin, 10 µM ultraglutamin and 5% (v/v) FBS (Lonza, Verviers, Belgium). In order to stimulate their differentiation into DC, cells were cultured in the presence of GM-CSF (50 ng/ml) and IL-4 (40 ng/ml). After 5 days of culture, immature DC were washed and seeded at 10^6^/well in 24-well plates. Stimuli – all in culture medium – were added to these DC and after 48 hours of culture supernatants were harvested for cytokine and chemokine profiling. For RNA profiling, RNA was isolated after 4 hours of culture. VSL#3, a mixture containing 8 different lactic acid bacteria, was purchased from Ferring Pharmaceuticals (Berkshire, UK). Freshly reconstituted bacteria from lyophilized stocks were added to DC cultures at a bacteria – DC ratio of 10 to 1. Ultrapure LPS *E. coli K12* (final concentration 1 µg/ml) was purchased from Invivogen (San Diego, California, USA).

### RNA isolation and transcription profiling using PCR array

Total RNA was isolated from 10^6^ cells using a RNAEasy Kit (Qiagen, Venlo, Netherlands) according to the manufacturer’s instructions. 500 ng RNA was used for single stranded cDNA synthesis using a High Capacity RNA-to-cDNA kit (Applied Biosystems, Carlsbad, CA USA) and incubated for 60 min at 37°C and 5 minutes at 95°C. cDNA was used for PCR-array analysis by using the RT^2^-Profiler PCR Array (human cytokine & chemokine array) from SuperArray Bioscience (Frederick, MD, USA). The PCR array combines the quantitative performance of SYBR Green-based real-time PCR with the multiple gene profiling capabilities of microarray. 96-well plates containing gene-specific primer sets for 84 chemokines and cytokines, 5 housekeeping genes, and 2 negative controls were used. For each experimental condition, cDNA from 6 individual donors was analyzed. Gene expression was normalized to internal controls (housekeeping genes) to determine the fold change in gene expression between test and control samples by ΔΔC_t_ (SuperArray Bioscience). The data discussed in this publication have been deposited in NCBI’s Gene Expression Omnibus and are accessible through GEO Series accession number GSE62583 (http://www.ncbi.nlm.nih.gov/geo/query/acc.cgi?acc=GSE62583).

### Chemokine profiling

Multiplex analysis was performed on the supernatants of DC for profiling of secreted chemokines (Invitrogen, human 10-plex) according to the manufacturer’s instructions. The beads were read on a LiquiChip 200, (Qiagen, Hombrechtikon, Switzerland), and data were analyzed by the five parameter curve fitting in Luminex100 IS Software. Chemokine concentrations are presented as pg/ml. Comparison with of multiplex bead array assays with ELISA assays showed comparable results with these two quantitative analysis methods (data not shown).

### NF-κB and STAT-1 phosphorylation assay

STAT-1 transcription factor activity was quantitatively detected by TransAM STAT family transcription factor assay (Active Motif, Carlsbad, CA) according to the manufacturer′s recommendations. In short, cell nuclei were isolated from DC using a nuclear extraction kit (Active Motif). After lysis of the nuclei, nuclear lysates (derived from 15*10^6^ cells) were incubated in 96-well dishes containing immobilized oligonucleotides containing a STAT consensus DNA-binding site (5′-TTCCCGGAA-3′). The specific primary antibody and the secondary antibody conjugated to horseradish peroxidase were incubated with the nuclear extracts following the vendor’s manual. The colorimetric reading (at 450 nm) was determined on a VERSAmax microplate. Nuclear extracts from COS-7 cells (treated with IFN-γ) were included as positive controls for STAT-1α. The phosphorylation assay for NF-κBp65 was performed as described for STAT-1, with slight modifications. Instead of TMB substrate, a chemiluminescent substrate was used to quantify phosphorylation of this transcription factor.

### Statistical Analysis

Statistical analyses were performed with the Rank-Wilcoxon paired test. Array data were analyzed using the Mann-Whitney U-test or Student’s t-test if the samples passed the normality analyses by using RT^2^ Profiler PCR Array Data Analysis. Unsupervised hierarchical clustering was performed using the web-based data tool of the manufacturer (SABioscience).

## Results

### LPS and VSL#3 induce distinct cytokine and chemokine profiles in human DC

We evaluated the modulation of TLR-4 induced signaling pathways in human DC by probiotic bacteria. DC from 6 individual donors were generated by culturing CD14^+^ monocytes for 5 days in the presence of GM-CSF and IL-4. Flow-cytometry analysis revealed that these DC had an immature phenotype, i.e. CD11c^+^CD86^low^CD86^low^CD83^low^. Stimulation of these immature DC with the TLR4 agonist LPS, the probiotic mixture VSL#3, or a combination of both resulted in a mature phenotype, evident from the enhanced expression of HLA-DR, CD86, and CD83 (data not shown).

**Figure 1:**
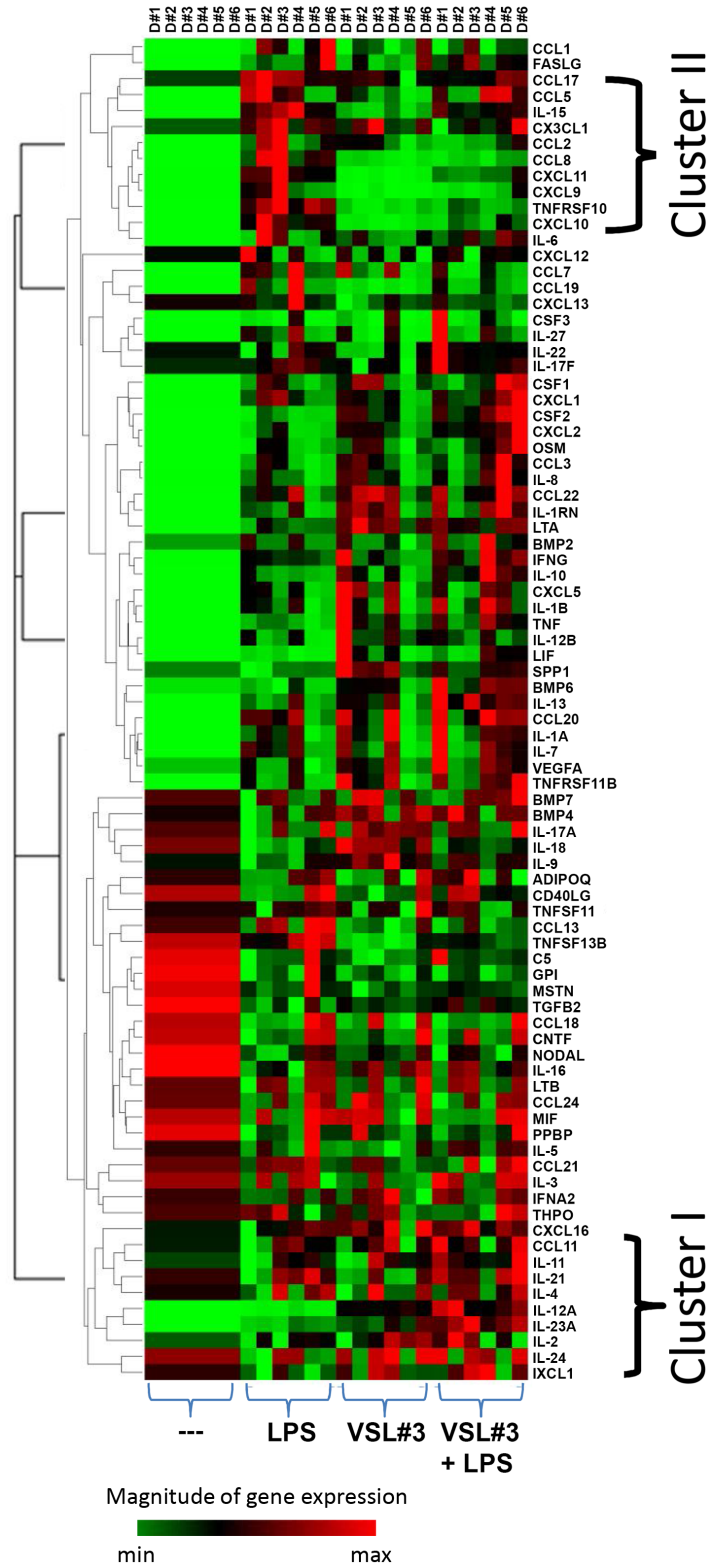
Chemokine and cytokine expression by stimulated DC. Clusterogram showing the hierarchical clustering of relative expression levels for all samples and all genes included in the real-time PCR arrays. Data for each gene were normalized to a panel of housekeeping transcripts. Relative expression levels for each individual gene are presented as minimum (green) and maximum (red). Rows represent gene expression profiles of individual samples, 4 hours after stimulation (6 donors for each experimental condition).

To assess the expression of chemokine and cytokines, RNA was isolated from DC left unstimulated or stimulated with LPS, VSL#3, or a combination of both, and subsequently analyzed with the use of a dedicated PCR gene array. A complete list of all 84 measured chemokines and cytokines and their fold induction relative to unstimulated DC is shown in Table 1. Transcripts of a large number of pro-inflammatory mediators were induced compared to immature DC, irrespective of the stimulus. However, the magnitude of induction or inhibition was gene- and stimulus-specific. Therefore, hierarchical clustering was applied to identify clusters of co-regulated genes. A heatmap showing the clustering of the expression patterns of 6 individual donors is depicted in Fig. 1. In this heatmap, minimum (green) and maximal (red) expression values are shown for each gene. Two major cluster could be identified as indicated in Fig. 1. Cluster I comprise genes that are moderately induced by LPS, compared to the mixture of bacteria. These include *IL-1α, IL-1β, CCL3, CSF2, CXCL2, IL-12A, IL-12B.* In addition, the co-incubation of human DC with VSL#3 and LPS synergistically induced *IL-23A* and *IL-12A* transcripts.

**Table 1:** mRNA expression levels of 84 chemokines and cytokines after 4 hours of stimulation with LPS, VSL#3 or a combination of both, as determined by qPCR arrays.

	Fold change (as compared to unstimulated cells)Fold change		
Chemokine/Cytokine	LPS	VSL#3	VSL#3+ LPS
*ADIPOQ*	1.4	1.2	1.4
*BMP2*	3.5	2.4	8.4
*BMP4*	1.1	1.9*	1.7*
*BMP6*	3.6	14.4**	52.3**
*BMP7*	−1.0	1.4	1.4
*C5*	−1.4	−1.8	−1.1
*CCL1*	762.7**	210.8*	234.3*
*CCL11*	1.6	1.8	2.7**
*CCL13*	2.0	−1.3	−1.1
*CCL17*	2.9**	1.9	2.3*
*CCL18*	1.0	−1.0	1.1
*CCL19*	45.7	4.4**	18.0
*CCL2*	45.6**	19.1*	16.2**
*CCL20*	954.5***	755.3*	1657.4***
*CCL21*	1.3	−1.1	1.1
*CCL22*	5.6**	8.1**	8.8**
*CCL24*	1.2	1.4	1.0
*CCL3*	47.7***	105.2**	159.5***
*CCL5*	412.7**	127.9*	476.7**
*CCL7*	6.8*	4.4	3.1
*CCL8*	145.8**	7.8	13.2***
*CD40LG*	−2.1	−2.7*	−1.3
*CNTF*	−1.4*	−1.4	−1.3
*CSF1*	14.9**	23.0**	26.9**
*CSF2*	182.0***	687.2*	2049.1***
*CSF3*	50.9	51.1	233.0
*CX3CL1*	4.5*	3.2	2.6
*CXCL1*	589.9**	403.5	1060.3**
*CXCL10*	8118.3***	217.6**	1488.8***
*CXCL11*	30832.8**	294.6***	7989.2**
*CXCL12*	1.8	1.3	1.7
*CXCL13*	1.4	−2.7*	−1.7
*CXCL16*	1.4*	2.0**	2.2***
*CXCL2*	153.5***	452.8	1088.3**
*CXCL5*	4.1*	7.9*	6.5*
*CXCL9*	4136.7*	42.5**	602.7**
*FASLG*	7.1	4.8**	10.2*
*GPI*	−1.4	−1.6*	−1.5*
*IFNA2*	−1.0	1.1	1.5
*IFNG*	41.5*	36.2	94.0*
*IL-10*	13.8	20.2	69.6
*IL-11*	1.8*	2.3	3.7*
*IL-12A*	73.4	1591.1*	3385.0*
*IL-12B*	1817.4*	9130.1*	5194.7**
*IL-15*	14.6*	2.7*	9.7***
*IL-16*	−10.6***	−6.2***	−8.2***
*IL-17A*	−1.0	1.4	1.3
*IL-17F*	2.0*	1.5	2.4**
*IL-18*	−2.7*	1.7*	−1.5
*IL-1A*	691.8*	925.2*	1771.6***
*IL-1B*	567.1*	895.1*	1560.5**
*IL-1RN*	8.4*	19.8***	25.2***
*IL-2*	2.1*	3.6*	3.5*
*IL-21*	1.3	−1.0	2.0**
*IL-22*	1.8	1.1	2.3
*IL-23A*	34.1*	104.4***	300.6*
*IL-24*	−1.2	1.3	1.3
*IL-27*	127.6*	27.5	144.4
*IL-3*	−1.0	−1.2	1.4
*IL-4*	1.4	1.2	1.7
*IL-5*	1.1	−1.4	1.4
*IL-6*	2184.1**	1657.6	3885.9
*IL-7*	142.2*	86.0	199.7*
*IL-8*	323.6***	632.7*	1090.3**
*IL-9*	−1.2	4.7	2.2
*LIF*	33.2***	246.7	284.0
*LTA*	57.2***	168.4***	157.7***
*LTB*	1.0	1.1	1.2
*MIF*	1.1	1.1	1.2
*MSTN*	−1.4	−1.9*	−1.3
*NODAL*	−2.4*	−2.3*	−2.5*
*OSM*	4.3***	7.4**	13.2*
*PPBP*	−1.4	−1.2	−1.2
*SPP1*	1.2	6.5***	4.6**
*TGFB2*	−3.1***	−2.5***	−1.6*
*THPO*	1.1	−1.1	−1.1
*TNF*	50.4***	206.5*	248.0***
*TNFRSF11B*	9.6*	18.0*	32.3***
*TNFSF10*	137.5***	6.9*	18.2***
*TNFSF11*	1.3	1.3	1.4
*TNFSF13B*	1.2	−3.6**	−1.9
*VEGFA*	2.8	7.7*	12.5*
*XCL1*	−1.5	1.5	1.9

Data for each gene were normalized to a panel of housekeeping transcripts and compared to unstimulated DC. Results represent the mean of 6 individual donors.

Results were statistically evaluated by Student’s t-test, comparing the replicate 2^Δct^ values for each gene in the control group and treatment groups.

*p<0.05, **p<0.01, and ***p<.0001.

**Figure 2:**
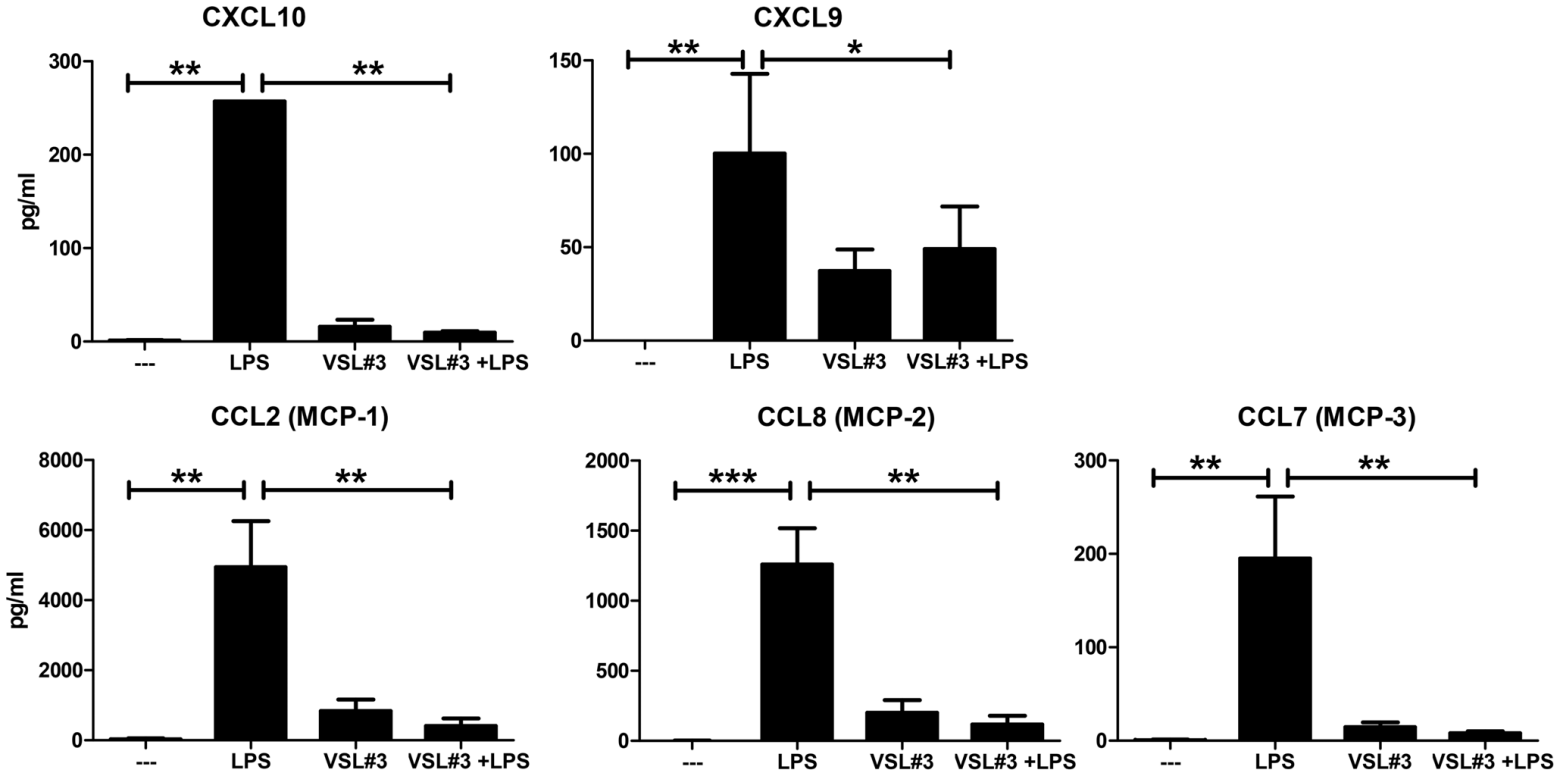
LPS-induced chemokine production of human DC is suppressed by VSL#3. A set of genes representing cluster II was confirmed at the protein level. Supernatants were harvested from replicate DC cultures after 48 hours after stimulation and performed in parallel with the cultures used for mRNA profiling. Levels of various cytokines and chemokines were determined by multiplex bead array technology. Results are expressed as pg/ml ± SD. Rank-Wilcoxon paired test: * p<0.05, ** p<0.01, *** p<0.0001.

We also identified a cluster of LPS-induced genes that were suppressed by VSL#3. This cluster (Fig. 1 cluster II) comprised *TNFRSF10* and a number of chemokines, including *CXCL9, CXCL10, CXCL11, CCL1, CCL2, CCL8*, and *CCL19*. These findings were confirmed on the protein level using a bead-array. As shown in Fig. 2, supernatants of replicate cultures performed in the presence of VSL#3 had diminished protein levels of CXCL9, CXCL10, CCL2, CCL7 and CCL8.

### VSL#3 inhibits LPS-induced phosphorylation of STAT-1

**Figure 3:**
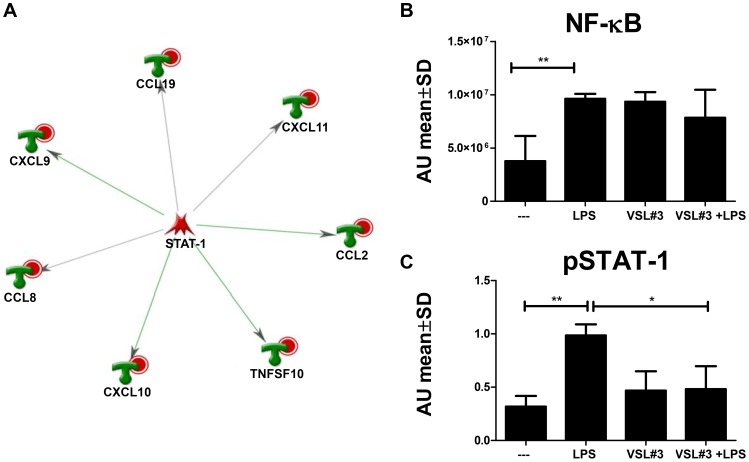
VSL#3 inhibits LPS-induced phosphorylation of STAT-1. (**A**) LPS-induced genes, significantly suppressed by VSL#3, were analyzed by using Metacore Network analysis (Transcription factor). STAT-1 was predicted to be a key driver of this cluster. DC were stimulated for 3 hours, and phosphorylation of NF-κBp65 (**B**) and STAT-1 (**C**) was quantified in nuclear extracts using the Trans-am assay. Absorbance values (mean ± SD) of 5 individual donors are shown. Rank-Wilcoxon paired test: * p<0.05, ** p<0.01.

Since only a distinct subset of LPS-induced genes was suppressed by VSL#3, we aimed to identity a common regulator for this cluster. *In silico* analysis using MetaCore software was used to identify transcription factors regulating genes in this cluster. Compared to the reference list of all 84 genes measured on the array, LPS-induced genes suppressed by VSL#3 were highly enriched for the transcription factor STAT-1 (z-score 173, p-value 1.3*10^−26^) as depicted in Fig. 3A.

To validate the role of STAT-1 in the suppression of LPS-induced gene expression found by this bioinformatics approach, an additional experiment was performed to measure phosphorylated STAT-1 in the nucleus upon stimulation. Therefore, DC were stimulated for 45 and 180 minutes before the isolation of nuclear proteins. Nuclear extract from 10^7^ DC was analyzed by TRANSam. This ELISA-based assay allows detection and quantification of DNA-binding activity of transcription factors, in this case pSTAT-1α and pNF-κBp65. NF-κBp65 was phosphorylated after 3 hours of incubation, irrespective of the stimulus (Fig. 3B). In contrast, pSTAT-1 was increased in nuclear extracts of DC after stimulation with LPS, but not if VSL#3 was present in cell culture (Fig. 3C). These data are in line with the *in silico* prediction, that LPS induced migration of STAT-1 to the nucleus upon phosphorylation was blocked in the presence of VSL#3.

## Discussion

We previously identified the modulation of chemokines as a major mechanism in the protective effect of the probiotic mixture VSL#3 in colitis [11]. Moreover, we showed that this probiotic mixture modulates chemokine expression *in* *vivo* in conditions of homeostasis (Mariman *et* *al*, submitted for publication). Dendritic cells are likely targets for these probiotic bacteria via binding to pattern recognition receptors [12], thereby modulating inflammatory signaling pathways [13]. The regulatory role of DC is of particular importance at mucosal surfaces as the intestine, where the immune system exists in intimate association with commensal bacteria [14]–[16]. In response to microbial signals, DC give rise to varying production levels of different cytokines and, accordingly, different effector functions [17], [18]. Indeed, modulation of chemokine expression by VSL#3 *in* *vivo* could partly be mimicked *in* *vitro* employing mouse bone marrow derived DC [19]. Subsequently, we wished to study if and how probiotic bacteria would modulate inflammatory responses in human DC.

Consistent with previous publications [20]–[22], probiotic lactic acid bacteria as well as the TLR4 ligand LPS were able to induce DC maturation. This maturation was accompanied by the enhanced expression of a large number of pro- and anti-inflammatory mediators. Robust responses with respect to cytokine expression are in line with previous reports in human and mouse DC [23], [24], and are related to high bacterial doses.

Interestingly, similar to our results obtained with BMDC derived from C57BL/6 [19], co-stimulation of human DC with VSL#3 and LPS synergistically induced *IL-23A* and *IL-12A* transcripts. Together with *IL-12B*, these subunits form IL-23 and IL-12p70, respectively. Secretion of these cytokines by DC may polarize naïve T-cells into T_h_17 and T_h_1 cells.

LPS can bind to TLR4 and thereby induce different signaling pathways. At the level of the plasma membrane, LPS activated TLR-4 binds to sorting adaptor (TIRAP), which then recruits the signaling adaptor myD88 to trigger the production of pro-inflammatory mediators [25]. However, activated TLR-4 in the endosomes engages the adaptor molecules TIRAP and TRAF to induce IFN-β and the anti-inflammatory IL-10 [26]. It has been reported that involvement of these two signaling pathways in LPS-induced chemokine expression varies considerably depending on the kind of chemokine [27]. CCL2, CCL7, CCL8, CXCL2, CXCL3, and CXCL9 largely rely on myD88, whereas CCL5, CXCL10 and CXCL13 are regulated by myD88 independent pathways. Since in this study VSL#3 inhibited both expression of myD88-dependent (CXCL9 and CCL8) and myD88-independent chemokines (CXCL10), it is tempting to conclude that VSL#3 does not interfere at the level of myD88- or TRIF-coupled TLR-signaling.

Numerous reports indicate an important role for the transcription factor STAT-1 in the regulation of inflammatory mediators [28]–[30]. Its particular role in chemokine expression was illustrated in *in* *vitro* and *in* *vivo* studies with STAT-1^−/−^ mice, showing the role of STAT-1 in LPS induced *Cxcl10* expression [31]. Furthermore, it was shown that blocking the JAK/STAT-1 pathway reduced the expression of *Cxcl9 and Cxcl10 in* IFN-γ-activated macrophages [32], [33]. Other model systems also highlighted the role of STAT-1, as shown in a study where mutations in the sequence of STAT-1 influenced IFN-γ induced expression of *Cxcl9*, *Cxcl10*, and *Ccl2* in HeLa cells [34]. Interestingly, these chemokines were present in the cluster of LPS-induced genes that were suppressed by VSL#3.

In view of these findings it was not surprising that transcription factor analysis using Ingenuity Software identified STAT-1 as a potential key regulator and downstream target of immunomodulation by VSL#3. Accordingly, we were able to demonstrate that VSL#3 specifically decreased the phosphorylation of this transcription factor, whereas phosphorylation of NF-κB was not suppressed (Fig. 3B). Although formal proof is required to substantiate a central role of STAT-1 in immunosuppression by VSL#3 and possibly also individual probiotic strains, further studies directed at signaling pathways that result in STAT-1 activation may be of great help to unravel part of the mechanism by which probiotic bacteria exert their immunosuppressive effect.

In conclusion, we showed that probiotic mixture VSL#3 interfere with LPS-induced STAT-1 phosphorylation in human DC in conjunction with the modulation of chemokine expression and secretion. This finding may contribute to a better understanding of the mechanisms by which this specific mixture exerts anti-inflammatory effects.
